# Cortical thickness abnormalities in trichotillomania: international multi-site analysis

**DOI:** 10.1007/s11682-017-9746-3

**Published:** 2017-06-29

**Authors:** Samuel R. Chamberlain, Michael Harries, Sarah A. Redden, Nancy J. Keuthen, Dan J. Stein, Christine Lochner, Jon E. Grant

**Affiliations:** 1 Department of Psychiatry, University of Cambridge, Box 189 Level E4, Addenbrooke’s Hospital, Cambridge, CB2 0QQ UK; 2 Cambridge and Peterborough NHS Foundation Trust, Cambridge, UK; 3 Department of Psychiatry & Behavioral Neuroscience, University of Chicago, Chicago, IL USA; 4 Department of Psychiatry, Massachusetts General Hospital and Harvard Medical School, Boston, MA USA; 5 MRC Unit on Anxiety & Stress Disorders, Department of Psychiatry, University of Cape Town, Cape Town, South Africa; 6 MRC Unit on Anxiety & Stress Disorders, Department of Psychiatry, University of Stellenbosch, Stellenbosch, South Africa

**Keywords:** Trichotillomania, Impulse, Impulsivity, Compulsivity, MRI, Neuroimaging

## Abstract

Trichotillomania is a prevalent but often hidden psychiatric condition, characterized by repetitive hair pulling. The aim of this study was to confirm or refute structural brain abnormalities in trichotillomania by pooling all available global data. De-identified MRI scans were pooled by contacting authors of previous studies. Cortical thickness and sub-cortical volumes were compared between patients and controls. Patients (*n* = 76) and controls (*n* = 41) were well-matched in terms of demographic characteristics. Trichotillomania patients showed excess cortical thickness in a cluster maximal at right inferior frontal gyrus, unrelated to symptom severity. No significant sub-cortical volume differences were detected in the regions of interest. Morphometric changes in the right inferior frontal gyrus appear to play a central role in the pathophysiology of trichotillomania, and to be trait in nature. The findings are distinct from other impulsive-compulsive disorders (OCD, ADHD, gambling disorder), which have typically been associated with reduced, rather than increased, cortical thickness. Future work should examine sub-cortical and cerebellar morphology using analytic approaches designed for this purpose, and should also characterize grey matter densities/volumes.

**Electronic supplementary material:**

The online version of this article (doi:10.1007/s11682-017-9746-3) contains supplementary material, which is available to authorized users.

## Introduction

Trichotillomania (also referred to as hair pulling disorder) is a psychiatric condition characterized by recurrent pulling out of one’s own hair (American Psychiatric Association 2013). Trichotillomania has an estimated lifetime prevalence of approximately 0.5–1%, and peak age of onset in adolescence (12–13 years of age) (Christenson et al. 1991; Cohen et al. 1995; Mansueto et al. 2007; Odlaug and Grant 2010). The condition is typically associated with impairment across domains of social, occupational, academic, and psychological functioning (Woods et al. 2006). Greater functional impairment is associated with worse symptom severity, later symptom onset, and with lower quality of life (Grant et al. 2016). Patients with trichotillomania can present across a range of medical settings, including to family doctors, dermatologists, neurologists, psychiatrists, pediatricians, endocrinologists, and geneticists (Couper 1999). Some patients with trichotillomania eat their own hair, which can lead to epigastric discomfort, and even bowel obstruction, due to the formation of trichobezoar or ‘hair balls’ (Frey et al. 2005).

Prevailing psychobiological models of obsessive-compulsive and related disorders emphasize the likely involvement of excessive drive from sub-cortical neural regions involved in habit generation (especially the dorsal striatum), coupled with a lack of top-down control from cortical regions involved in mediating habit suppression (Grant and Chamberlain 2016). Of all disorders characterized by repetitive behavior, trichotillomania is uniquely situated as a candidate transspecies model of a mental disorder: hair pulling is a relatively specific type of observable behavior and is seldom driven by intrusive thoughts or cognitions (in contrast to those behaviors seen in obsessive-compulsive disorder [OCD]). Furthermore, hair pulling can be seen as an extreme form of grooming behavior, which is also observable in many animal species. Candidate animal models of excessive grooming abound (for discussion see Camilla d'Angelo et al. 2014). For example, mice with knockout of the hoxb8 gene – which is involved in neuronal development – show excessive grooming resulting in loss of hair and skin damage (akin to human trichotillomania and skin picking disorder, respectively) (Greer and Capecchi 2002). The hoxb8 gene is ordinarily expressed in mice orbitofrontal cortex and striatum, regions also heavily implicated in the pathophysiology of OCD in humans (Chamberlain et al. 2007; Fouche et al. 2017).

Despite the suggestion that trichotillomania might fit within a neurobiological model involving dorsal striatum and cortical abnormalities, very few imaging studies have been conducted in individuals with this condition. The available studies have used relatively small sample sizes, and have also used a variety of methodological techniques, which makes it difficult to draw firm conclusions. In part, this probably reflects limited funding availability for less well known mental disorders such as trichotillomania. Published individual imaging studies of trichotillomania have had sample sizes of 9–21 patients and 10–19 controls. Even with a sample size of 21 versus 19, power would have been limited to identify significant group differences, even those differences with large effect size. Small sample sizes can also increase the risk of individual studies detecting statistically significant findings that are not true positives. One potential means of overcoming these limitations, used with recent success in the context of other disorders, is so-termed ‘mega-analysis’, in which findings from disparate studies of a given disorder are pooled to yield overall effects (Fouche et al. 2017). Surface-based morphology is potentially advantageous over more traditional imaging methods, and enables the highly sensitive characterization of cortical thickness. Therefore, the aim of the current study was to obtain available MRI scans (along with select demographic and clinical information) from research groups that have published peer-reviewed data papers of trichotillomania; and to use FreeSurfer software to examine cortical thickness and sub-cortical structure volumes between patients and controls in the pooled dataset. We hypothesized that trichotillomania would be associated with cortical thickness abnormalities in frontal cortical sectors coupled with excess volume of the dorsal striatum (putamen/caudate). We further hypothesized that this abnormality would not correlate with symptom severity in patients, supporting its trait-like nature.

## Materials and methods

### Literature search and data collection

A literature search was conducted using PubMed to identify all published structural imaging studies of individuals with trichotillomania as of 1st February 2017 ([“MRI” or “imaging” or “magnetic resonance”] + [“trichotillomania” or “hair pulling” or “hairpulling”]. PubMed began in 1996 and the earliest trichotillomania MRI paper we found in the extensive literature search was from 1997. Reference lists from key review papers were also manually screened for additional source papers (Chamberlain et al. 2009; Flessner et al. 2012; Grant and Chamberlain 2016; Johnson and El-Alfy 2016). The authors of MRI-related trichotillomania publications were asked to contribute de-identified data. Data collection and sharing was approved by Institutional Review Boards covering the respective research sites; participants at each site provided informed, written consent. Raw de-identified MRI scans were shared plus the following select demographic/clinical measures for each patient and controls: age, gender, level of education, medication status, and (for patients) symptom severity based on the Massachusetts General Hospital Hair Pulling Scale (MGH-HPS) (Keuthen et al. 2007a), a self-report measure of trichotillomania symptoms for the past week.

### Data analysis

Between-group differences in demographic and clinical measures were explored using independent sample t-tests (*p* < 0.05, two-tailed, uncorrected), using SPSS v22.0.

Imaging pre-processing and data extractions were undertaken on the University of Chicago Midway computing system. MRI scans were processed using FreeSurfer software (surfer.nmr.mgh.harvard.edu) a methodology that has previously been validated (Dale et al. 1999; Reuter et al. 2012). In brief, scans for each subject were standardized, bias-field corrected, and skull-stripped. Cortical surfaces were identified using automated algorithms, normalized to the standard Freesurfer template, and smoothed using a standard 10 mm full width half maximum (FWHM) kernel. Volumes of a priori sub-cortical structures of interest were also extracted using Freesurfer’s parcellation techniques (caudate, putamen, nucleus accumbens, and hippocampus). Cortical thickness was compared between the trichotillomania and control groups, across the whole cortical surface, using the Qdec interface, with a significance threshold of voxel-wise *p* < 0.001 and cluster-wise *p* < 0.05, Monte Carlo corrected (10,000 simulations). Potential group differences in the volumes of sub-cortical structures were explored by exporting subject-level data from Freesurfer into SPSS v22.0, and conducting independent sample t-tests with significance defined as *p* < 0.05 uncorrected (this being exploratory). Correlation analysis (Spearman’s r) was used to explore relationships between any neural abnormalities that were identified in the preceding steps and symptom severity (*p* < 0.05 uncorrected). Effects of study site (research team), and psychotropic medication, on any neural abnormalities were explored using analysis of covariance and independent sample t-tests.

## Results

The pooled sample comprised a total of 76 adults with trichotillomania and 41 healthy controls. Data were supplied from four research groups (see supplementary online file for sample sizes and other details for each site). Other research groups that were contacted either were unable to provide data due to prospective data sharing consent not being taken or did not reply to correspondence. This applied to two studies, one conducted in the USA and one in the Republic of Korea.

The mean (standard deviation) disease severity in the trichotillomania group was 15.6 (4.7) on the MGH-HPS, consistent with (on average) mild-moderate severity. Groups did not differ significantly in terms of age, gender, education levels, total grey volume, or estimated total intracranial volumes (all *p* > 0.10; Table 1). The majority of patients (*N* = 70, 92.1%) were un-medicated. Patients who were medicated were taking: citalopram (*n* = 1), fluoxetine (*n* = 1), sertraline (*n* = 2), venlafaxine (*n* = 1), bupropion (*n* = 1).

**Table 1:** Demographic and clinical characteristics of the pooled sample

	Trichotillomania cases	Healthy Controls	Statistic	*p*-value
	(*n* = 76)	(*n* = 41)		
Age (mean, SD)	33.43 (11.67)	32.42 (10.76)	0.46	0.64
MGH-HPS total score	15.6 (4.7)	N/A	N/A	N/A
Gender (n, %)			c^2^ = 0.58	0.45
Male	6 (7.9%)	5 (12.2%)		
Female	70 (92.1%)	36 (87.8%)		
Education (n, %)			c^2^ = 0.11	0.95
Standard only	8 (10.5%)	5 (12.5%)		
College/lower degree	17 (22.4%)	9 (22.5%)		
Graduate/higher degree	51 (67.1%)	26 (65.0%)		
Estimated total grey matter volume, mm^3^	607,888.1 (53,954.4)	609,362.3 (56,068.6)	−0.14	0.89
Estimated total intracranial volume, mm^3^	1,459,464 (142,631.8)	142,631.8 (204,211.9)	0.88	0.38

c^2^ = chi-square

**Fig. 1:**
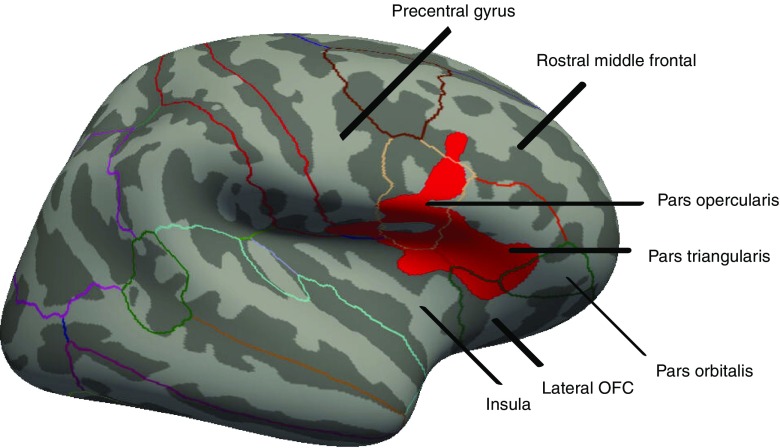
Cluster of significantly increased cortical thickness in trichotillomania versus healthy controls (in red), superimposed onto standard Freesurfer template (thin coloured lines indicate Freesurfer labelled brain divisions). The peak co-ordinates were in the pars triangularis, with the cluster also extending into the other labelled brain regions

Compared to controls, those with trichotillomania showed significantly increased cortical thickness in a cluster at the right inferior frontal gyrus (Fig. 1, voxel-wise *p* < 0.001, cluster-wise *p* < 0.05 Monte-Carlo corrected). The cluster was of extent 1679.1mm^2^, cluster-wise p (CWP) = 0.0007, with peak co-ordinates in the right pars triangularis [Talairach co-ordinates: 37.7, 24.5, 7.1]. Extracted mean cortical thickness in this cluster was 2.59 mm (0.18 mm) in trichotillomania cases, and 2.45 mm (0.20 mm) in controls. Medicated and un-medicated patients did not differ significantly in terms of cortical thickness in this cluster [un-medicated 2.59 mm (0.18 mm), medicated 2.51 mm (0.17 mm), *p* = 0.269]. The group difference in cortical thickness remained statistically significantly after controlling for study site (scanner type) using analysis of covariance (*p* < 0.001), and there was no main effect of study site (scanner type) (*p* = 0.504). Cortical thickness in this cluster did not correlate significantly with MGH-HPS total scores in patients (Spearman’s rho −0.10, *p* = 0.391). The trichotillomania and control groups did not differ significantly from each other in terms of volumes of sub-cortical structures of interest (Table 2).

**Table 2:** Volumes of a priori sub-cortical structures of interest in patients with trichotillomania and healthy controls. No between-group findings approached statistical significance

	Mean volume (standard deviation), mm^2^			
Subcortical region	Trichotillomania cases (*N* = 76)	Healthy Controls (*N* = 41)	Statistic (t)	*p*-value
Left				
Caudate	3602.10 (411.56)	3602.53 (456.02)	−0.006	0.99
Putamen	5625.71 (805.38)	5639.71 (1101.97)	−0.072	0.94
Thalamus	7500.75 (834.58)	7265.08 (678.12)	1.552	0.12
Nucleus Accumbens	600.36 (126.78)	635.76 (171.030	−1.164	0.25
Hippocampus	4074.03 (518.96)	4074.45 (533.64)	−0.004	0.99
Right				
Caudate	3726.71 (430.58)	3740.54 (479.54)	−0.159	0.87
Putamen	5376.90 (749.94)	5489.32 (903.61)	−0.719	0.47
Thalamus	7209.13 (793.86)	7006.09 (593.96)	1.434	0.15
Nucleus Accumbens	575.50 (103.6)	595.56 (144.13)	−0.788	0.43
Hippocampus	4155.00 (494.41)	4165.12 (468.52)	−0.108	0.91

## Discussion

This study represents the largest analysis of structural brain differences between adults with trichotillomania and healthy controls. Based on an analysis of pooled raw MRI data obtained from research groups that had published at least one previous peer-reviewed imaging paper in patients, we found that trichotillomania was associated with significantly increased cortical thickness in a right frontal cluster, with its peak in the pars triangularis – part of the right inferior frontal gyrus. The extent of abnormality was unrelated to symptom severity, and was unaffected by research site. Reduced right frontal cortical thickness has previously been reported in mega-analysis of structural studies in OCD (Fouche et al. 2017), highlighting that the neural substrates of trichotillomania and OCD diverge markedly (excess thickness in the former; diminished thickness in the latter), despite both disorders long considered similar and now being in the same diagnostic category in DSM-5. These divergent findings suggest that efforts to understand the neurobiology of OCD based on transspecies models of trichotillomania may be misplaced.

The right inferior frontal gyrus constitutes an important node in cortico-subcortical circuitry involved in the top-down suppression of inappropriate motor responses, based on multiple tiers of evidence from studies using go/no-go and stop-signal cognitive paradigms (for detailed discussion, see ^26^) (for discussion see Aron et al. 2014). People with focal damage to the right inferior frontal gyrus show response inhibition deficits, the magnitude of which correlates significantly with volume of damage specifically to this area (Aron et al. 2003). Disruption to right inferior frontal gyrus via transcranial magnetic stimulation impairs response inhibition on the stop-signal task (Chambers et al. 2006). In a large sample of adolescents, activation in a right frontal network was related to the ability to suppress pre-potent motor responses on a stop-signal task; and was also related to variation in a norepinephrine/noradrenaline reuptake transporter gene (Whelan et al. 2012). The finding of abnormal cortical thickness in the right inferior frontal gyrus in trichotillomania may help to account for neuropsychological findings with regards to this disorder. Statistically significant inhibitory control deficits have been reported in most but not all studies of trichotillomania, versus healthy controls. Interestingly, pilot data suggest that this stop-signal deficit extends to clinically asymptomatic first-degree relatives of patients (Odlaug et al. 2014). As to the nature of increased cortical thickness in this region in trichotillomania, there are several possibly explanations. This could represent aberrant cortical pruning over time in patients; or a more inherent abnormality perhaps relating to genetic predisposition. It seems unlikely to be accounted for by compensatory brain changes arising from hair pulling itself, given that this region was also abnormal in unaffected first-degree relatives in a prior study. However, there could be additional changes to this region over time due to hair pulling itself. These issues cannot be resolved in the context of the current study design.

Previous neuroimaging findings in trichotillomania were inconsistent across studies. Most previous studies examined grey matter volumes or densities. Volume/density based studies variably reported the following grey matter differences in patients compared to controls: reduced left striatal (putamen) volumes (O'Sullivan et al. 1997); reduced cerebellar volumes (Keuthen et al. 2007b); increased striatum, amygdalo-hippocampal formation, and cortex [cingulate, supplementary motor, frontal] densities (Chamberlain et al. 2008); or reduced left inferior frontal and increased right cuneal volumes (post hoc) (Grachev 1997). One study found no significant group volumetric differences in the regions of interest (Stein et al. 1997). One study examined cortical thickness and found excess thickness in patients versus controls in the right inferior/middle frontal cortex, right lingual gyrus, left temporal cortex, and left precuneus (Odlaug et al. 2014). The current pooled analysis, including data from many of the above studies, focused on cortical thickness rather than volumes/densities. Measurement of cortical thickness is somewhat distinct from measurement of density/volume, because density/volume quantification using voxel-based morphometry may reflect not only cortical thickness but also differences in scan intensity, surface area, and/or cortical folding. As such, the current findings should not be seen as automatically refuting earlier density/volume findings – we intend to analyze these more traditional measures in future work using the same dataset. Rather, these data highlight a key role for excess thickness in the inferior frontal gyrus in the pathophysiology of trichotillomania, in support of a key finding from earlier work (Odlaug et al. 2014).

Several limitations should be considered in relation to the current study. Because we relied on historical data being supplied by research groups, the scope of measures available for analysis was restricted. For example, there was no access to data regarding historical treatments – only current medications. We were not able to assess the impact of duration of symptoms / age at symptom onset, nor of comorbidities, because this information was not available. Only six patients were receiving psychotropic medications at the time of study participation, hence the current study was not powered to identify effects of medication (if any) on brain structure in patients. Notably, the excess right frontal cortical thickness in the trichotillomania patients remained significant when the six medicated individuals were excluded, rigorously demonstrating that this key result was not driven by medication confounds. Lastly, Freesurfer provides only crude, partial measures of cerebellar morphology and as such our study chose not to examine this structure, as we anticipate this could be examined in future work using more specialist software.

In summary, trichotillomania was associated with significantly elevated cortical thickness in the right inferior frontal gyrus compared to matched healthy controls. This morphometric abnormality appears to be trait in nature, because it was unrelated to symptom severity, and prior pilot data have identified similar results in asymptomatic first-degree relatives of trichotillomania patients. Contrary to expectation, no volumetric abnormalities of sub-cortical structures of interest were found. This may indicate that previous studies, which have reported sub-cortical basal ganglia abnormalities in trichotillomania, may have only applied to certain manifestations of the disorder, or constituted false positive results (Grant and Chamberlain 2016). Future work should examine the three-dimensional morphology of sub-cortical structures in trichotillomania, as well as of the cerebellum, because this may reveal additional abnormalities associated with the disorder not detected herein. It would also be valuable to use functional imaging, as there is a dearth of such papers in the trichotillomania literature to date.

## Electronic supplementary material

ESM 1 (DOCX 21 kb)
