# Supplementary material for: Cortical thickness abnormalities in trichotillomania: international multi-site analysis
Source: Brain Imaging Behav. 2017 Jun 29;12(3):823–8. doi: 10.1007/s11682-017-9746-3 (PMC5640149; doi:10.1007/s11682-017-9746-3)
Supplement: Supplementary file 1 — (DOCX 21 kb) [file 11682_2017_9746_MOESM1_ESM.docx]

**Supplementary Online File – Chamberlain et al. – Trichotillomania (TTM) mega-analysis**

**Table 1. Summary of data sources**

|  |  | N of provided scans suitable for data analysis | | Criteria for TTM | Medication status | Co-morbidities | Scanner type, and other relevant details |
| --- | --- | --- | --- | --- | --- | --- | --- |
| Laboratory Source | Relevant previous publication(s) | TTM | Controls |  |  |  |  |
| Lochner | Roos A. et al. A comparison of brain volume and cortical thickness in excoriation (skin picking) disorder and trichotillomania (hair pulling disorder) in women. Behav Brain Res*.* 15;279:255-8. Additional scans provided from on-going studies. | 17 | 15 | DSM-IV trichotillomania, right handed, no lifetime or current psychiatric disorder, no significant physical or neurological illness, no current pregnancy, stabilized pharmacotherapy regimen for last 3 months, if any | All un-medicated but 4 of TTM subjects | One patient had comorbid ADHD, GAD and specific phobia, four patients had comorbid specific phobia, one patient had comorbid compulsive shopping, one patient had comorbid BDD and self- injurious behavior | 3T Siemens Allegra |
| Keuthen | Keuthen NJ et al. Evidence for reduced cerebellar volumes in trichotillomania. Biol Psychiatry. 61(3):374-81; Rauch SL et al. Brain activation during implicit sequence learning in individuals with trichotillomania. Psychiatry Research - Neuroimaging, 154 (3): 233-240. | 14 | 12 | DSM-IV trichotillomania, no comorbid OCD or other psychiatric conditions, no lifetime diagnoses of psychiatric illness, hair pulling symptoms for longer than 4 months, no psychotropic medications in last 4 weeks | Un-medicated | One TTM patient had GAD, 2 HC had specific phobias | 1.5T Sonata |
| Chamberlain | Chamberlain SR et al. Grey matter abnormalities in trichotillomania: morphometric  magnetic resonance imaging study. The British Journal of Psychiatry, 193(3), 216–221. | 20 | 19 | DSM-IV trichotillomania; no significant depression or OCD; no prior neurological conditions; no treatment for TTM for preceding 6 months. | Un-medicated | One patient had comorbid panic disorder and agoraphobia; the others were free from current Axis I comorbidities | 1.5 T GE Signa system |
| Grant | Odlaug BL et al. Impaired response inhibition and excess cortical thickness as candidate endophenotypes for trichotillomania. *Journal of Psychiatric Research*. 2014;59:167–173 | 12 | 14 | DSM - 5 trichotillomania, no contraindication to MRI, no pregnancy, no history of bipolar, dementia or psychotic disorder, no initiation of behavior therapy or psychotropic medications within last 6 months, no past 12 months SUD | 2 were medicated | One patient had comorbid MDD, one patient had comorbid GAD and MDD | 3 T Phillips Achieva Quasar dual 16 Ch system |

Abbreviations: TTM = trichotillomania, T = Tesla, ADHD = attention deficit hyperactivity disorder, GAD = generalized anxiety disorder, HC = healthy controls, OCD = obsessive-compulsive disorder, MDD = major depressive disorder, SUD = substance use disorder, MRI = magnetic resonance imaging.

**Table 2a** Demographic and clinical data from Lochner Laboratory (South Africa)

|  | TTM Subjects  (n=30) | Healthy Controls  (n=0) |
| --- | --- | --- |
| Age (mean, SD) | 35.80 (13.63) | n/a |
| Gender (n, %)  Male  Female | 4 (13.3%)  26 (86.7%) | n/a |
| Education (n, %)  Completed standard  education but not beyond  College/lower degree  Graduate/higher degree | 7 (23.3%)  7 (23.3%)  16 (53.3%) | n/a |
| MGH Score (mean, SD) | 14.28 (4.92) | n/a |
| Number on current medication | 4 (13.3%) | n/a |

MGH = Massachusetts General Hospital Hairpulling Scale

**Table 2b** Demographic and clinical data from Keuthen Laboratory (U.S.A.)

|  | TTM Subjects  (n=14) | Healthy Controls  (n=11) |
| --- | --- | --- |
| Age (mean, SD) | 29.07 (6.86) | 29.27 (7.66) |
| Gender (n, %)  Male  Female | 0 (0%)  14 (100%) | 0 (0%)  11 (100%) |
| Education (n, %)  Completed standard  education but not beyond  College/lower degree  Graduate/higher degree | 0 (0%)  4 (28.6%)  10 (71.4%) | 3 (27.3%)  3 (27.3%)  5 (45.5%) |
| MGH Score (mean, SD) | 17.86 (3.84) | - |
| Number on current medication | 0 | 0 |

MGH = Massachusetts General Hospital Hairpulling Scale

**Table 2c** Demographic and clinical data from Chamberlain Laboratory (United Kingdom)

|  | TTM Subjects  (n=20) | Healthy Controls  (n=20) |
| --- | --- | --- |
| Age (mean, SD) | 37.50 (11.54) | 32.80 (8.91) |
| Gender (n, %)  Male  Female | 3 (15%)  17 (85%) | 2 (10%)  18 (90%) |
| Education (n, %)  Completed standard  education but not beyond  College/lower degree  Graduate/higher degree | 0 (0%)  2 (10%)  18 (90%) | 1 (5.3%)  3 (15.8%)^  15 (78.9%)^ |
| MGH Score (mean, SD) | 15.10 (4.34) | - |
| Number on current medication | 0 | 0 |

MGH = Massachusetts General Hospital Hairpulling Scale

**Table 2d** Demographic and clinical data from Grant Laboratory (U.S.A.)

|  | TTM Subjects  (n=12) | Healthy Controls  (n=10) |
| --- | --- | --- |
| Age (mean, SD) | 25.83 (4.51) | 35.10 (16.18) |
| Gender (n, %)  Male  Female | 0 (0%)  12 (100%) | 2 (20%)  8 (80%) |
| Education (n, %)  Completed standard  education but not beyond  College/lower degree  Graduate/higher degree | 1 (8.3%)  4 (33.3%)  7 (58.3%) | 1 (10%)  3 (30%)  6 (60%) |
| MGH Score (mean, SD) | 17.08 (5.01) | - |
| Number on current medication | 2 (16.7%) | 0 |

MGH = Massachusetts General Hospital Hairpulling Scale
